# Hemoptysis secondary to rupture of infected aortic aneurysm– a case report

**DOI:** 10.1186/s13019-019-0959-y

**Published:** 2019-07-25

**Authors:** Hina Inam, Ibrahim Zahid, Sarim Dawar Khan, Salman Ul Haq, Saulat Fatimi

**Affiliations:** 1 Cardiothoracic Surgery, Aga Khan University Hospital, Karachi, Pakistan; 2 Dow University of Health Sciences, Karachi, Pakistan; 3 Aga Khan University Hospital, Karachi, Pakistan

**Keywords:** Aortic aneurysm, Rupture, Hemoptysis, Infection

## Abstract

**Background:**

Massive hemoptysis is a life-threatening condition and can arise as a complication of various conditions. It rarely occurs as a complication of a ruptured thoracic aortic aneurysm. Even rarer are conditions where pseudoanurysms of aorta result due to infection.

**Case presentation:**

A 30 year-old female patient presented with left sided chest pain, intermittent fever, cough and massive hemoptysis. A pseudo-aneurysm of proximal descending thoracic aorta at the level of the left Subclavian artery was noted over CT scan.

Upon performing a left posterolateral thoracotomy, the aneurysm was seen to have ruptured into the apical segment of left upper lobe, contained mainly by a thrombus. The anterior wall of the pseudoaneurysm was debrided and a bovine pericardial patch was used to repair the aortic defect. Cultures of the tissue obtained showed Enterobacter species, therefore the patient was prescribed 6 weeks of IV antibiotics following surgery. Post-operative CT scan revealed reduced diameter of the aorta. She was discharged in good health and remains well at follow up evaluation.

**Conclusions:**

We present a case of hemoptysis caused by a ruptured descending aorta aneurysm into left lung. The aneurysm was secondary to infection by Enterobacter. Surgical repair of the concerned region of aorta was effective, without any major sequelae. To the best of our knowledge, no such cases have been reported previously.

## Introduction

Customarily, hemoptysis can result from a number of diseases like tuberculosis, bronchiectasis, pulmonary embolism, aspergilloma and bronchial carcinoma [1]. Rarely, rupture of an aortic aneurysm into lungs may also lead to hemoptysis. Aortic aneurysms are a common entity in the ascending and descending part but isolated aortic aneurysms are relatively rare [2]. Even rarer conditions are infective pseudoaneurysms of the aorta, commonly due to *Staphylococcus aureus* and *epidermidis*, *Streptooccus* and *Salmonella*. Such aneurysms have a high morbidity and mortality [3], and are known as mycotic aneurysms.

We hereby report a case of ruptured infected aortic aneurysm presenting with hemoptysis.

## Case

**Fig. 1:**
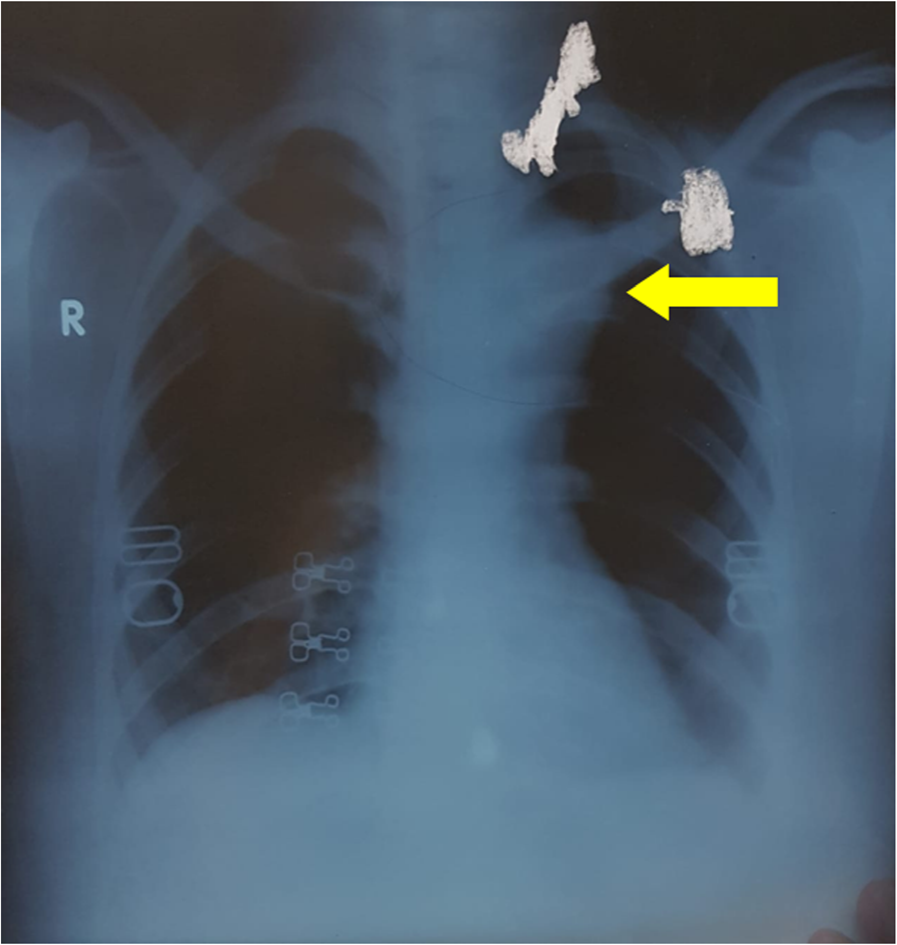
Chest Xray PA view showing widened mediastinum (yellow arrow)

**Fig. 2:**
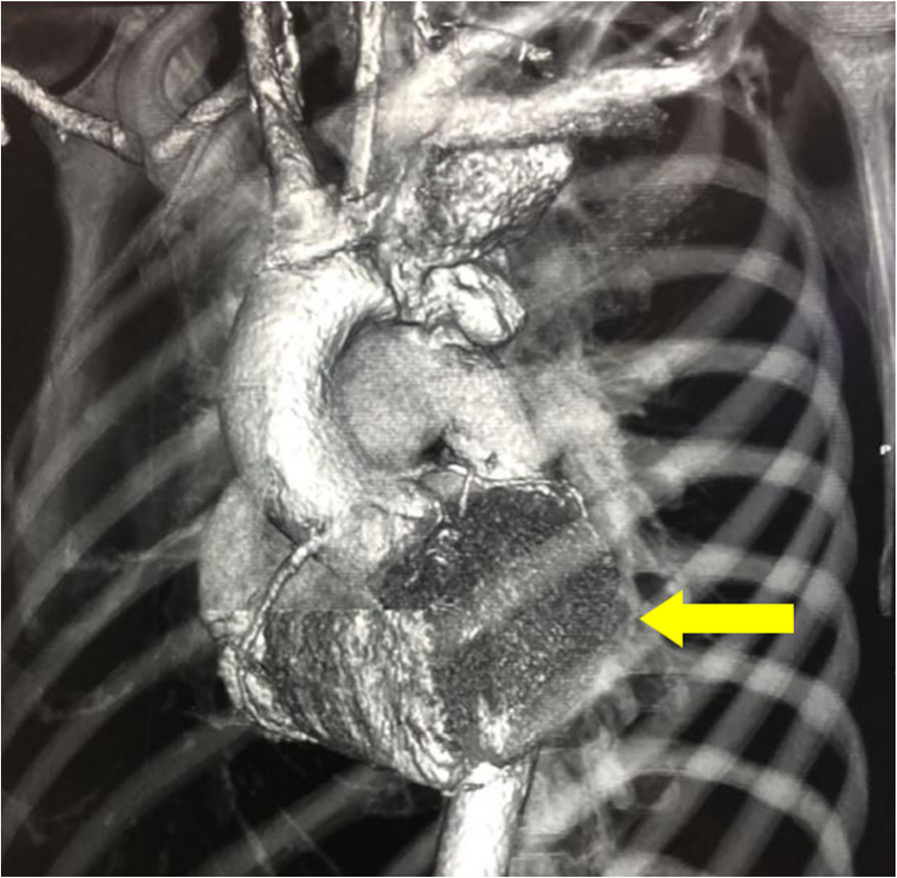
A reconstructed CTA showing the position and relative size of the aneurysm in the descending aorta (yellow arrow) near left subclavian artery

**Fig. 3:**
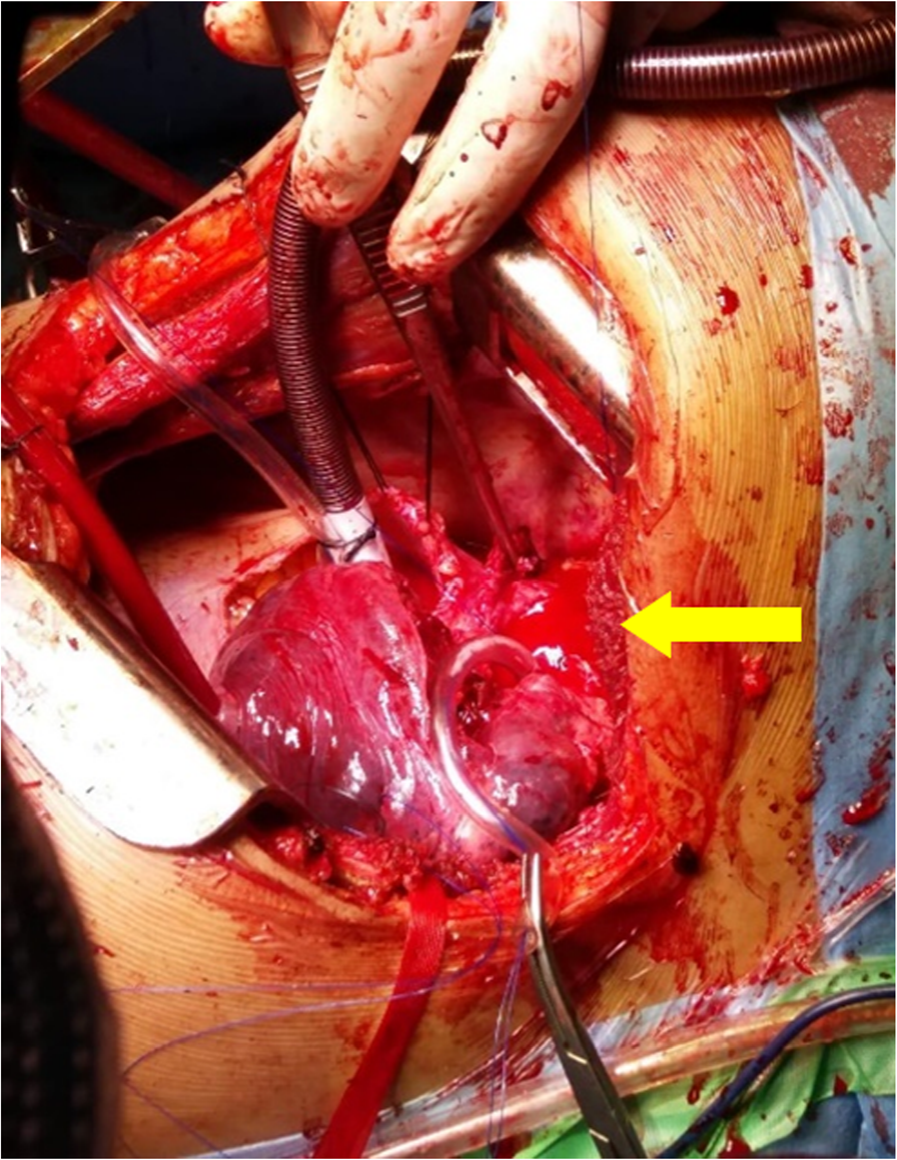
Intra-operative picture showing a ruptured aortic anreuysm (yellow arrow)

A 30 year-old female patient presented with left sided **chest pain** and intermittent **fever** and cough for 6 months and massive **hemoptysis** for a week. There was a history of cerebral venous sinus thrombosis in the sigmoid sinus about 2 years ago for which she received Rivaroxaban. Chest X-ray revealed widened mediastinum (Fig. 1). Upon investigating through CT scan, pseudo-aneurysm of **proximal descending thoracic aorta** at the level of the left Subclavian artery was found (Fig. 2); the diameter was 83 mm, with a patent lumen of 33 mm. A partially occluding thrombus was also present, as seen in Fig. 3. Consequently the patient was admitted and Internal Medicine team was consulted preoperatively to rule out mycotic aneurysm. Serology including AMA, ANA, VDRL, IgG and IgM were all negative; however an ESR of 90 was obtained.

Subsequently, an urgent repair of the aneurysm was planned. Left posterolateral thoracotomy was performed and the large pseudo-aneurysm of the descending aorta from the origin of the subclavian artery was identified. The aneurysm was seen to have ruptured into the apical segment of left upper lobe leading to intermittent hemoptysis and was contained mainly from the thrombus.

With partial bypass via main pulmonary artery and distal aortic cannulation, proximal and distal control was obtained and the aneurysm was opened. The posterior wall of the pseudo-aneurysm was intact and the anterior wall was debrided. A bovine pericardial patch was used to repair the aortic defect; multiple plegetted sutures were also used to strengthen the repair. The patient was taken off bypass and after successful hemostasis, the chest was closed. Post operative course was un remarkable. The aortic wall, left lung tissue, and para-aortic lymph node samples were sent for histopathological analysis, bacterial, fungal and TB culture and sensitivity. They showed fibrocollagenous tissue fragments with necrotic inflammatory exudate and the lymph nodes showed reactive lymphoid hyperplasia. The cultures grew Enterobacter species sensitive to Amikacin, Meropenem and Imipenem, confirming the diagnosis of an infected aortic aneurysm. The patient was hence, prescribed IV Imipenem for 6 weeks. She continues to follow up in clinic and is in good health.

## Discussion and conclusions

Hemoptysis can result from a number of underlying conditions. Massive hemoptysis can generally be fatal resulting in asphyxia from airway obstruction and exsanguinations, and it accounts for a mortality rate of 30–70% [4]. Due to the uncertain nature of the clinical course, it is necessary to investigate the causative pathology for suitable treatment. Nevertheless occasionally rare causes of hemoptysis like a ruptured aortic aneurysm might not be considered initially as a possible differential, deferring the timely recognition and management of the disease. Similarly, in this case, where the presenting symptoms were chest pain and fever, hemoptysis can be a rare manifestation which could be massive; since a thrombus was containing the aneurysm here, the hemoptysis was minimal and there was no resultant hemodynamic instability. Hemoptysis is fairly common in thoracic aortic dissection but ruptured aortic aneurysms rarely cause the same. Literature reveals a homogenous report however the aneurysm was found in the abdomen and into the lower lobe of right lung [5]. On the other hand, our case showed the aneurysm rupturing into the upper lobe of left lung.

Common etiologies of aortic aneurysm include infection, atherosclerosis, cystic medial necrosis, Marfans and Ehler-Danlos syndrome which can cause weakening of the aortic wall. The inflammation in the aneurysm wall could either be caused due to infection of the aneurysm spreading to the lung tissue or from stasis in bronchioles due to compaction of lung parenchyma from the aneurysm and resultant infection affecting the aneurysm wall. The infection weakens the wall, causing it to eventually rupture. According to a recent report, majority of the thoracic aneurysms were found to be atherosclerotic in nature, while only 4% had an infective cause [6]. Usually infected aortic aneurysms occur in patients who are immunocompromised like those with diabetes mellitus, liver cirrhosis, end-stage renal disease, alcoholism, chronic glucocorticoid therapy, chemotherapy, post transplantation immunosuppression, human immunodeficiency virus infection, and malignancy [7]. Infection of the aorta can result from a number of Gram-positive and negative bacteria. Salmonella and *Staphylococcus aureus* are considered the two most common agents [8]. The Enterobacter species identified in our case is typically uncommon here.

There are four main types of vascular infections with infected aneurysms in accordance with the Wilson’s classification [9]

1. aneurysm formation after microbial arteritis due to bacteremia or local infection invasion,
2. posttraumatic infected pseudo aneurysms, which were usually related to drug abuse in the past and with increased incidence with the use of endovascular procedures
3. infection of preexisting aneurysms, and
4. infected (mycotic) aneurysm resulting from infective endocarditis-related septic emboli

Arterial wall intima is generally resistant to infections however it may become susceptible to infections due to an injury or any pathological change. Untreated infection may result in abscesses, perforations and pseudo aneurysms [9].

For diagnostic purposes, transesophageal echocardiography is a useful technique in evaluating various aortic pathologies like dissection, aneurysm and rupture [10]. However, the stepwise approach mainly includes two noninvasive tests, Chest X ray and CT scan. Our case demonstrates how the two techniques help in identification of an opacity and diagnose the rupture into the adjacent lung. A combination of these investigations can diagnose rupture of an aneurysm into lungs 63% of the times [11].

While investigating into the problem is simple here, the consequent management can vary based on the clinical condition of the patient. In case of life threatening hemoptysis, it is important to stabilize the patient hemodynamically following the maintenance of airway and improving oxygenation. Ultimately, the patient has to be managed by either placing an endovascular stent-graft or repairing the aneurysm. The trend of endovascular management of such ruptures has recently increased as a minimally invasive procedure. It helps in stopping hemorrhage without invasive surgery in the elderly, with significant co-morbids and risk factors. A decline has been documented in the immediate morbidity and mortality from the procedure in recent studies, as compared to open procedure [12]. However there are several risks associated; the caliber of affected area should be adequate enough to support the stent, administration of intravenous contrast can cause allergy and it can adversely affect kidneys, and in certain instances, unavailability of graft and stent in case of an emergent procedure can pose problem [13].

Therefore the most suitable method of treatment for an infected thoracic aortic aneurysm is the resection and repair/bypass of the affected region [14]. Chances remain that the infection might recur following surgical correction, so Long et al. proposes a combination of surgical as well as medical management [15] with prolonged antibiotic course. Mostly, the procedures are performed while on cardio-pulmonary bypass along with circulatory arrest. Periaortic tissue is widely excised, followed by debridement of infected aorta and abscesses. Nonetheless non-traumatic aortic rupture overall has poor prognosis, with postoperative mortality ranging up to 50% due to the high incidence of severe surgical risk factors [16].

Where endovascular repair has been shown to significantly decrease the 30-day mortality when compared to open surgery [17, 18], Doss et al. [19] reported that, in contrast to open surgery, benefits of endovascular treatment declined over time due to a significant rise in the number of complications especially neurologic, and reoperations. Therefore, endovascular repair is better considered as a palliative bridging technique to control of hemorrhage during the acute phase so that patients can recover sufficiently to undergo definitive surgical repair [12]. When the blood loss is not massive, an elective open surgery remains the best option conclusively.

## Data Availability

The datasets used and/or analysed during the current study are available from the corresponding author on reasonable request.

## Abbreviations

AMA: Anti-mitochondrial antibody
ANA: Ant-nuclear antibody
CT scan: Computed Tomography
ESR: Erythrocyte Sedimentation Rate
IV: Intravenous
VDRL: Venereal Disease Research Laboratory test
